# TIM-3 ameliorates host responses to *Salmonella* infection by controlling iron driven CD4^+^ T cell differentiation and interleukin-10 formation

**DOI:** 10.1016/j.ebiom.2025.105910

**Published:** 2025-09-11

**Authors:** Christa Pfeifhofer-Obermair, Natascha Brigo, Chiara Volani, Piotr Tymoszuk, Egon Demetz, Sabine Engl, Günter Weiss

**Affiliations:** a Department of Internal Medicine II, Medical University of Innsbruck, Anichstr. 35, Innsbruck 6020, Austria; b Christian Doppler Laboratory for Iron Metabolism and Anemia Research, Medical University of Innsbruck, Anichstr. 35, Innsbruck 6020, Austria

**Keywords:** TIM-3, Iron, Intracellular bacteria, IL-10, IFNgamma, CD4 T cells

## Abstract

**Background:**

Iron loading increases infection risk in being a nutrient for invading siderophilic bacteria and by modulating immune functions including the expression of the immune checkpoint regulator T-cell immunoglobulin-and-mucin-containing-domain-3 (TIM-3). TIM-3 affects specific immune cell functions including T-helper cell differentiation but also T cell dysfunction, and immune exhaustion. Given the prevalence of iron overload specifically in patients at higher risk for infection such as those suffering from hemo-oncological diseases, we investigated TIM-3’s role in immune control of bacterial sepsis.

**Methods:**

A sepsis model was employed in wildtype and *Tim3*^*−/−*^ mice with transgenic expression of a functional natural resistance associated macrophage protein 1 (NRAMP1). This creates a chronic inflammation model with enhanced resistance to infections with Gram negative *Salmonella typhimurium*, enabling to study T cell immune responses over time.

**Findings:**

Dietary iron supplementation reduced mouse survival, which was further exaggerated by TIM-3 deletion. This indicates that TIM-3 dependent immune regulation was essential for effective host defence against *Salmonella*. TIM-3 deletion increased the production of immune-deactivating interleukin (IL) −10 as a result of impaired interleukin-12 receptor (IL-12R)-dependent CD4^+^ cytotoxic T cell signalling and development which subsequently reduced the production of anti-microbial interferon gamma (IFNγ). Anti-IL-10 treatment in iron-loaded *Tim3*^*−/−*^ mice improved *Salmonella* control and restored CD4^+^ T cell mediated IFNγ production.

**Interpretation:**

Our study uncovers TIM-3 as a crucial regulator of T cell driven immune control of bacterial infection and identifies the underlying treatable pathways, which is of major importance to better combat infection related mortality in subjects with iron overload syndromes.

**Funding:**

Christian-Doppler-Society, 10.13039/501100002428FWF (I-3321, W-1253).

**Research in context**

**Evidence before this study**

In chronic infections and cancer T cells are faced by continuous antigenic stimulation which leads to immune exhaustion. This is characterised as a state of cellular and immunological dysfunction with over-expression of a distinct set of inhibitory cell surface receptors, one of which is T-cell immunoglobulin and mucin containing domain 3 (TIM-3). While the role of TIM-3 in mediating T cell exhaustion has been largely established in models of cancer, autoimmunity or viral infections little evidence is available on the potential importance of TIM-3 in bacterial sepsis. Iron overload is a frequent clinical condition, which can result from genetic diseases or transfusional iron accumulation in hematological/oncological/transplanted patients. Those subjects are at a high risk for infection related morbidity and mortality, and this can be further aggravated by enhanced iron availability for siderophilic microbes. Given that iron affects helper T cell 1 (Th1) differentiation via modulation of TIM-3 expression and that *Salmonella*-specific CD4^+^ Th1 cells are crucial for effective host response against these pathogens, the uncovering of the role of TIM-3 in the immune control of bacterial infection under iron loading conditions would generate important information for the treatment of patients at risk.

**Added value of this study**

Employing a sepsis model in NRAMP1-expressing C57BL/6 mice and TIM-3 knockout mice infected with the intracellular bacterium *Salmonella typhimurium* we found that dietary iron supplementation resulted in a shortened survival of wildtype mice, which was further drastically reduced upon genetic deletion of TIM-3. We demonstrated that TIM-3 mediated crucial immune effector pathways to control *Salmonella* infection in an iron-rich environment. TIM-3 deletion increased IL-10 production due to impairment of IL-12R-dependent CD4^+^ cytotoxic T cell signalling and differentation along with decreased formation of the anti-microbial effect cytokine IFNγ. IL-10 neutralisation in iron loaded Tim3^−/−^ mice resulted in improved *Salmonella* infection control and restorage of CD4^+^ mediated IFNγ formation.

**Implications of all the available evidence**

Our study uncovers TIM-3 as a crucial regulator of T cell driven immune control of bacterial infection and identifies the underlying treatable pathways, which is of major importance to better combat infection related mortality in subjects with iron overload syndromes.

## Introduction

Iron is a key component of several enzymatic processes and thus mammalian cells require iron for metabolism and proliferation, apart from the importance of iron for haem synthesis and oxygen transport. Most iron for the daily needs is provided by macrophages through recycling of the metal from senescent red blood cells.^1^^,^^2^ Furthermore, iron is also a crucial nutrient for the multiplication and pathogenicity of most microbes. Therefore, iron homoeostasis has to be tightly regulated, specifically during infection and inflammatory processes.^3^

The uptake of iron via transferrin receptor-1 (TfR-1) is crucial for lymphocyte proliferation and differentiation.^4^ In addition, iron availability controls effector functions and cytokine formation of macrophages and T-cells *in vitro* and *in vivo*, thereby triggering infection outcomes to a myriad of pathogens.^5^ Iron overload is a frequent clinical condition, which can result from genetic diseases or transfusional iron accumulation in hematological/oncological/transplanted patients. Those subjects have been shown to have a higher morbidity and mortality risk for infection with siderophilic microbes.^6^ Upon activation by various signals naïve CD4^+^ T cells proliferate and differentiate into specific subsets including T-helper cells type 1 (Th1), Th2, Th17, Th9, Th22, and regulatory T cells (Treg), which then produce a typical set of cytokines.^4^ Among those, interferon-gamma (IFNγ) plays a central role in the control of innate and adaptive immune responses. IFNγ is mainly secreted by T cells, especically, T-helper cells type 1, natural killer cells (NK cells) and natural killer T cells (NKT cells). The release of IFNγ is essential for host defence against infection with intracellular microbes by regulating adaptive immune function and anti-microbial immune effector pathways by antigen presenting cells (APCs) and macrophages.^7^^,^^8^ After binding to its receptor the Janus kinase (JAK) signal transducer and the transcription protein (STAT) pathways are activated resulting in expression of a myriad of anti-microbial effector molecules and cytokines.^9^

In chronic infections and cancer T cells are faced by continuous antigenic stimulation. This promotes the generation of exhausted T cells. Exhaustion is characterised as a state of cellular and immunological dysfunction. Exhaustion leads to a reduction of cytokine production accompanied by an over-expression of a distinct set of inhibitory cell surface receptors like programmed cell death protein 1 (PD-1), cytotoxic T-lymphocyte-associated Protein 4 (CTLA-4), Lymphocyte-activation gene 3 (Lag-3), and T cell immunoglobulin and mucin containing protein-3 (TIM-3).^10^ Specifically, TIM-3 is crucial in the regulation of various immune cell functions.^11^^,^^12^ However, at the same time, TIM-3 acts as a key factor in T cell dysfunction and exhaustion.^13^^,^^14^ While the role of inhibitory receptors such as TIM-3 in mediating T cell exhaustion has been largely established in models of cancer, autoimmunity or viral infections^10^ little evidence is available on the potential importance of TIM-3 in bacterial sepsis. In mice infected with *Mycobacterium tuberculosis,* TIM-3 expression was associated with impaired immune control of infection via T cell exhaustion.^15^

*Salmonella enterica* serovar *typhimurium* (*S. typhimurium*) is a Gram negative, intracellular bacterium, the murine correlate to human *Salmonella typhi*, causing severe systemic, potentially fatal infection in mice. Being a siderophilic bacterium, *Salmonella* relies heavily on iron. Genetic factors and excessive dietary iron promote bacterial growth and weakens the immune response, resulting in a reduced control of the infection.^16^ Moreover, several studies showed a crucial role for *Salmonella*-specific CD4^+^ Th1 cells as an important host response mechanisms to these pathogens.^8^ Given that iron affected Th1 differentiation via modulation of TIM-3 expression^17^ we were interested to further characterise the role of TIM-3 in the control of infection under iron loading conditions.

Here, we provide evidence that detrimental effects of high systemic iron on the integrity of bacterial immune defence in a model of systemic *S*. *typhimurium* infection can be traced back to an iron-mediated induction of the anti-inflammatory cytokine IL-10 when TIM-3 is absent. This is linked to a reduced expression of the signalling-responsible IL-12Rβ2 chain of the IL-12R and reduced production of protective IFNγ by CD4^+^ T cells. Administration of anti-IL-10 to iron-loaded *Tim3*^*−/−*^ animals or to splenocytes *ex vivo* restored IFNγ expression of CD4^+^ T cells and improved infection control under high iron conditions. This indicates that the negative checkpoint modulator TIM-3 acts as a crucial, iron-dependent regulator of T cell immune response to control expansion of intracellular *Salmonella*.

## Methods

### Mice

Nramp^G169^ C57BL/6 wildtype and conventional Nramp^G169^ C57BL/6 *Tim3*^*−/−*^ mice had free access to food and water and were housed according to institutional and governmental guidelines in the animal facility of the Medical University of Innsbruck with a 12-h light-dark cycle and an average temperature of 20 °C ± 1 °C. Animal experiments were approved by the Austrian Federal Ministry of Science and Research (licence number BMWF-66.011/0113-WF/V/3b/2016, 2020-0.659.059, 2022-0.344.278) according to the directive 2010/63/EU. Nramp^G169^ C57BL/6 wildtype mice were a kind gift from Ferric C. Fang (University of Washington, Seattle). *Tim3*^*−/−*^ Balb/c mice were kindly provided by Takeda Pharmaceuticals International Co. *Tim3*^*−/−*^ Balb/c mice were backcrossed to Nramp^G169^ C57BL/6 background for ten generations. All experiments were performed with male mice.

### Infection of mice

Wildtype *S. enterica* serovar *typhimurium* (*S*. *typhimurium*) strain ATCC14028 was used for experiments and grown under sterile conditions in Lysogeny broth (LB) medium (Sigma–Aldrich; 2024 Merck KGaA, Darmstadt, Germany) to a late-logarithmic growth phase and quantified using a cell counter and analyser (CASY, 45 μm capillary, OMNI Life Science, Bremen, Germany; RRID:SCR_002080). For infection experiments, male mice were fed with different iron diets for two weeks prior and during the course of the infection. Low iron diet had an iron content of ≤ 9 mg iron/kg diet,^18^ high iron diet contained 5 g iron/kg diet (both diets from Altromin; Lage, Germany). Male mice (8–12 weeks) were infected intraperitoneally with 1000 living S*. typhimurium* bacteria in 200 μl PBS (Lonza; Cologne, Germany).^19^ After 11 days of infection mice were euthanised by cervical dislocation, blood, spleens and livers were isolated, erythrocytes lysed (ACK buffer; 150 mM NH_4_Cl, 10 mM KHCO_3_, 0.1 mM Na_2_EDTA; Sigma–Aldrich; 2024 Merck KGaA, Darmstadt, Germany), and flow cytometry was performed (schematic overview). The bacterial load of organs was determined by plating serial dilutions of organ homogenates on LB agar plates (Sigma–Aldrich; 2024 Merck KGaA, Darmstadt, Germany) under sterile conditions and the number of bacteria per gram of tissue was calculated. Mouse survival data were analysed with a Cox regression and the Kaplan–Meier method using Wilcoxon’s test.

**Figure:**
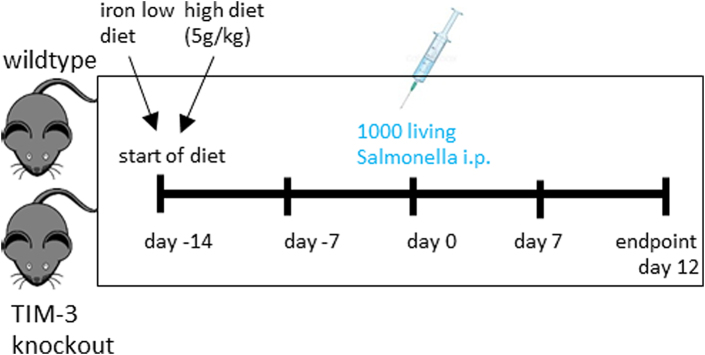

Schematic overview of the infection model.

### IL-10 blocking antibody experiments

Mice were fed and infected as described. Additionally, at day 9 post-infection, mice were intraperitoneally injected once with 100 μg inVivo MAb anti-mouse IL-10 antibody (BioXCell; BIOZOL Diagnostica, Eching, Germany; BE0049) or InVivoMAb rat IgG1 isotype control (BioXCell; BIOZOL Diagnostica, Eching, Germany; BE0088) in 200 μl PBS (Lonza; Cologne, Germany).

### Flow cytometry analysis

Spleens were homogenised through a 100 μm nylon cell strainer (Falcon) and red blood cells were lysed by incubation in ACK buffer (150 mM NH_4_Cl, 10 mM KHCO_3_, 0.1 mM Na_2_EDTA; Sigma–Aldrich; 2024 Merck KGaA, Darmstadt, Germany) for 2 min at room temperature. Flow cytometry staining was performed with panels of antibodies specific for T cells (anti-CD45-FITC BioLegend Cat# 147710, RRID:AB_2563542, anti-CD3-Biotin BioLegend Cat# 100243, RRID:AB_2563946 + Streptavidin-PeCy7, anti-CD4-FITC BioLegend Cat# 100405, RRID:AB_312690, anti-TIM-3-APC BioLegend Cat# 119706, RRID:AB_2561656, anti-PD1-PE, CXCR3-FITC BioLegend Cat# 126535, RRID:AB_2566564, IL12Rβ2-PE, CX3CR1-PE BioLegend Cat# 149020, RRID:AB_2565703) and neutrophils, macrophages and monocytes (anti-CD45-FITC BioLegend Cat# 147710, RRID:AB_2563542, anti-F4/80-BV421 BioLegend Cat# 123132, RRID:AB_11203717, anti-CD11b-APC BioLegend Cat# 101211, RRID:AB_312794, anti-Ly6G-PerCPeF710, anti-MerTK-PECy7, anti-Ly6C-BV510), in PBS with 0.5% FBS 2 mM EDTA for 15 min.

For intracellular staining cells will be stimulated with a mix containing 10 μg/ml Brefeldin A (Sigma–Aldrich; 2024 Merck KGaA, Darmstadt, Germany), 50 ng/ml PDBu (Sigma–Aldrich; 2024 Merck KGaA, Darmstadt, Germany) and 500 ng/ml ionomycin (Sigma–Aldrich; 2024 Merck KGaA, Darmstadt, Germany) in RPMI-1640 (PAN Biotech; Aidenbach, Germany) plus 10% FBS (Biochrom, Vienna, Austria) plus 1% penicillin/streptomycin (Lonza; Cologne, Germany) plus 2 mM L-glutamine (Lonza; Cologne, Germany) for 4 h prior to FACS staining. Brefeldin A leads to the blockade of protein transport to the Golgi complex and therefore the accumulation of proteins in the endoplasmic reticulum. Cytokines are trapped inside the cells and can be detected by intracellular staining. The cells were then formalin-fixed, permeabilized (0.05% Triton X-100 in PBS) and stained for cytokines (anti-IFNγ-PE, anti-IL17-FITC, anti-IL4-PE, anti-IL10-PE), and transcription factors (anti-FOXP3-FITC) for 1 h. All antibodies were from Biolegend (Amsterdam, The Netherlands). Cells were analysed with a Cytoflex S flow cytometer (Beckman Coulter, Krefeld, Germany) and FlowJo Software (Beckton Dickinson, Heidelberg, Germany, RRID:SCR_008520).

### Heat inactivation of Red Fluorescent Protein (RFP)-expressing *Salmonella*

Red Fluorescent Protein (RFP)-expressing *S. enterica* serovar *typhimurium* strain SL1344^20^ was used for experiments and grown under sterile conditions in LB medium (Sigma–Aldrich) to a late-logarithmic growth phase. The bacteria were centrifuged for 10 min at 4500 rpm and washed twice with PBS (Lonza; Cologne, Germany) and the pellet was resuspended in 20 ml PBS (Lonza; Cologne, Germany). Heat inactivation was performed in a water bath for 20 min at 70 °C. The amount of bacteria was quantified using a cell counter and analyser (CASY, 45 μm capillary, OLS Life Science, Bremen, Germany, RRID:SCR_002080), protein levels were quantified using a Bradford assay and stock concentrations of 1 mg/ml were stored at −80 °C.

### Cultivation of splenocytes on cell culture inserts

Mice were infected as described. At day 11 spleens were isolated and 1.2 × 10^8^/ml splenocytes were cultured on cell culture inserts (Thermo Fisher Scientific Inc, Vienna, Austria) in 6 well plates (Corning, Berlin, Germany) (500 μl cells plus 500 μl medium in the cell culture inserts, 2 ml medium in the 6 well). RPMI-1640 medium (PAN Biotech; Aidenbach, Germany) supplemented with 10% FBS (Biochrom, Vienna, Austria), 2% sodium pyruvate (Sigma–Aldrich; 2024 Merck KGaA, Darmstadt, Germany), 1× non-essential amino acids (Gibco, Thermo Fisher Scientific Inc, Vienna, Austria), 0.01% β-mercaptoethanol (Roth, Krems, Austria), 1% penicillin/streptomycin (Lonza; Cologne, Germany) and 2 mM L-glutamine (Lonza; Cologne, Germany) was used. Cells were re-stimulated with Red Fluorescent Protein (RFP)-expressing heat inactivated *Salmonella* (10 μg/ml) and, were indicated, anti-IL10 (Amsterdam, The Netherlands) was added at a concentration of 10 μg/ml. As negative control heat-inactivated wildtype *S*. *typhimurium* strain ATCC14028, which does not express RFP was used. After 48 h, cells were analysed by flow cytometry. For the characterisation of macrophages with engulfed (RFP)-expressing heat inactivated *Salmonella* we used a panel containing anti-CD3-FITC (BioLegend Cat# 100204, RRID:AB_312661), anti-CD19-FITC (BioLegend Cat# 152404, RRID:AB_2629813), anti-CD49b-FITC (BioLegend Cat# 103504, RRID:AB_313027), anti-CD11b-BV650, anti-CD45-APCR700, anti-F4/80-BV421 (BioLegend Cat# 123132, RRID:AB_11203717), and anti-Ly6G-PerCPeF710.^21^ All antibodies were from Biolegend. Cells were analysed with a Cytoflex S flow cytometer (Beckman Coulter, Krefeld, Germany) and FlowJo Software (Beckton Dickinson, Heidelberg, Germany, RRID:SCR_008520).

### RNA extraction and quantitative real-time PCR

Total RNA was prepared from nitrogen-frozen tissues with peqGOLD Tri-Fast™ (VWR, Dresden, Germany). For reverse transcription 4 μg RNA was used. Real-time PCR was performed on a CFX96 light cycler (Bio-Rad Laboratories GmbH, Vienna, Austria) using Ssofast Probes Supermix and Ssofast EvaGreen Supermix (Bio-Rad Laboratories GmbH, Vienna, Austria). Relative gene expression was calculated with the ΔΔCT method, normalising the results to the value for the Hypoxanthine phosphoribosyltransferase (*Hprt*) gene. *Hprt* fw 5′-gaccggtcccgtcatgc-3′, rv 5′-tcataacctggttcatcatcgc-3′, probe 5′-acccgcagtcccagcgtcgtc-3′; *Il-10* fw 5′-ccagagccacatgctcctaga-3′, rv 5′-tggtcctttgtttgaaagaaagtct-3′, probe 5′-tgcggactgccttcagccagg-3′; *Tgfβ* fw 5′-tgacgtcatggagttgtacgg-3′, rv 5′-ggttcatgtcatggatggtgc-3′, probe 5′-ttcagcgctcactgctcttgtgacag-3′.

### Seahorse

Mice were infected as described. At day 11 CD4^+^ T cells were isolated from spleens using a MagniSort™ Mouse CD4 T cell Enrichment Kit (Invitrogen, Cat# 8804-6821-74).

Freshly isolated CD4^+^ T Cells were counted, a minimum of 2.5 × 10^6^ cells/ml were frozen in FBS containing 10% DMSO using CoolCell freezing device. Cells were kept at −80 °C until the mitochondrial analysis. Mitochondrial functional analysis was performed using a Seahorse XF HS Mini Analyser (Agilent, Vienna, Austria) as described in.^22^ On the day of the mitochondrial functional analysis, frozen cell vials were quickly thawed in the water bath, resuspended in). RPMI-1640 medium (PAN Biotech; Aidenbach, Germany) supplemented with 10% FBS (Biochrom, Vienna, Austria), 1% penicillin/streptomycin (Lonza; Cologne, Germany) and 2 mM L-glutamine (Lonza; Cologne, Germany) and centrifuged for 7 min at 250 g at RT. Cell pellet was the resuspended in 500 μl of the assay medium, based on Seahorse XF DMEM Medium, pH 7.4 (Agilent, 103575-100), containing Seahorse XF Glucose 10 mM (Agilent, 103577-100), Seahorse XF Pyruvate 1 mM (Agilent, 103578-100) and Seahorse XF L-Glutamine 2 mM (Agilent, 103579-100). Cells were counted using the LUNA-FL™ Dual Fluorescence Cell Counter system (BioCat, Heidelberg, Germany). 2 × 10^5^ cells/well were seeded in a Seahorse XFp Cell Culture Miniplates, previously coated with 30 μl Poly-D-Lysine (0.1 mg/ml, Gibco A3890401) for 1 h at RT, washed 3 times with water and air-dried.

Seahorse XFp Cell Culture Miniplates were centrifuged at 200 g, acceleration 5, brakes off/slow brakes, for 2 min at RT to adhere cells to the bottom of the well. A total of 180 μl of assay medium was present in all wells, including well A and H, which were used as background. Cells were incubated in a non-CO2 37 °C incubator for around 45 min. XFp sensor cartridges were hydrated overnight with the appropriate calibrant solution (Seahorse XF Calibrant Solution) and loaded with the reagents to perform MitoStress test. Briefly, respiration of intact cells was measured as follows: (1) ROUTINE/BASAL respiration corresponding to the physiological respiration, (2) LEAK respiration was determined by the addition of Oligomycin (Omy, 1 μM), (3) maximal respiratory capacity was determined by two titrations of the protonophore Carbonyl cyanide 4-(trifluoromethoxy)phenylhydrazone (FCCP, 1 μM each step, final concentration 2 μM), (4) injection of complex I inhibitor rotenone (Rot, 0.5 μM) and complex III inhibitor antimycin A (Ama, 0.5 μM) led to the assessment of the residual oxygen consumption (ROX).

For data representation, oxygen consumption rate (OCR) was corrected for ROX (the value of the ROX respiration was subtracted to each step).

### Statistics

Statistical analysis was generated using Prism GraphPad software Version 10 (RRID:SCR_002798). For multiple comparisons two-way Analysis of variance (ANOVA) combined with Tukey’s post hoc test were performed. The survival curve was analysed with a Log-rank (Mantel–Cox) test. p values less than 0.05 were considered as statistically significant in any test.

### Ethical approval statement

All animal experiments were approved by the Austrian Federal Ministry of Science and Research (licence number BMWF-66.011/0113-WF/V/3b/2016, 2020-0.659.059, 2022-0.344.278) according to the directive 2010/63/EU. Nramp^G169^ C57BL/6 wildtype and conventional Nramp^G169^ C57BL/6 *Tim3*^*−/−*^ mice were housed in standard cages with a maximum density of 4 animals per cage. All mice had free access to food and water and environmental enrichment and were housed according to institutional and governmental guidelines in the animal facility of the Medical University of Innsbruck with a 12-h light-dark cycle and an average temperature of 20 °C ± 1 °C. To standardise the experiments related to iron metabolism, only male mice were are used. Animals were randomly allocated to groups, and the group sizes were determined based on extensive prior experience with comparable experimental setups. Sample size calculations were done with GPower 3.1. (ANOVA effect size f 0.2, α err 0.05, power 0.8) with the primary outcome *Salmonella* CFU. For all experiments three independent replicates were performed. For infections mice were injected intraperitoneal (i.p.) by always the same investigator. Daily health assessments were conducted by another investigator, and moistened food was placed on the cage floor to help maintain hydration and provide nutritional support. Daily body weights and body surface temperature measurement were obtained. Animals were euthanised at specific, pre-established time points following deep anaesthesia induced by i. p., injected ketamine (100 mg/kg) - xylazine (10 mg/kg). Humane endpoints were applied following a Grimace-Score (https://www.nc3rs.org.uk/mouse-grimace-scale) and a score table based on a scoring system already established for experiments or this kind.^23^

### Role of funders

The funders had no role in study design, data collection, analysis and interpretation, writing and submission of the manuscript. All authors had complete access to the data.

## Results

### The lack of TIM-3 decreases anti-bacterial host defence in iron loaded mice infected with *Salmonella*

Based on our previous observations that iron regulates Th1 T cell differentiation via upregulation of TIM-3 in an inflammation model driven by *Salmonella typhimurium (S. typhimurium)*^17^ we hypothesised an influence on anti-bacterial host defence in mice where TIM-3 is absent.

To this end, we used C57BL/6 wildtype mice (wt) and TIM-3 knockout (*Tim3*^*−/−*^) mice with transgenic expression of a functional natural resistance associated macrophage protein 1 (NRAMP1 or SlC11A1), which results in an improved host resistance to infections with Gram negative *S. typhimurium*.^24^ Functional NRAMP1 allows a prolonged bacterial infection, thus creating a chronic inflammation model, which allows to study T cell immune responses over a longer period of time.

**Fig. 1:**
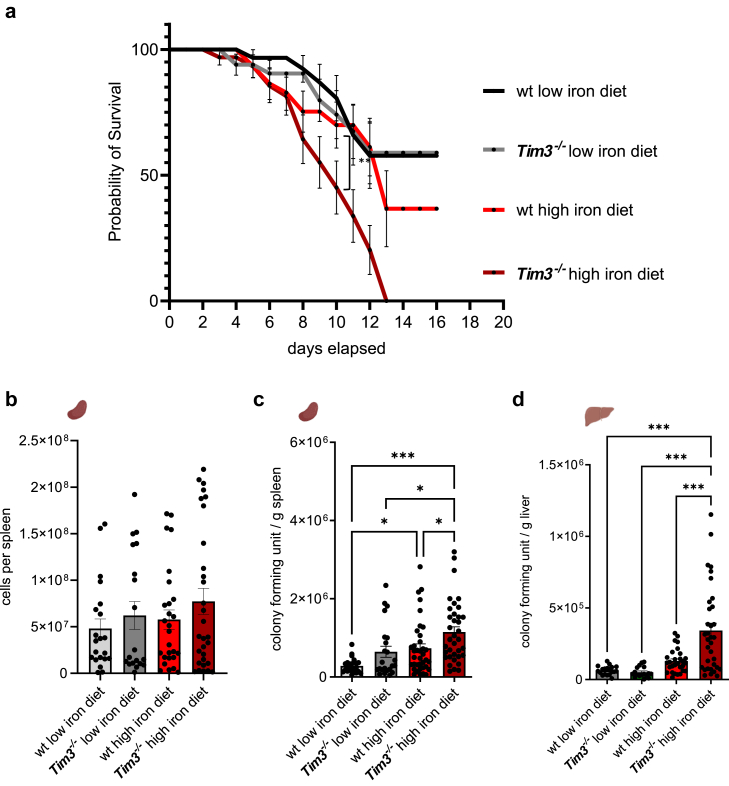
**Dietary iron supplementation decreases anti-bacterial host defence of *Tim3*^*−/−*^ mice in a long-term *Salmonella typhimurium* infection model.** Mice were fed different iron diets for two weeks prior and during the course of an intraperitoneal infection with 1000 living *S*. *typhimurium*. (a) Survival was analysed until day 16. (b) Total number of cells per spleen was determined by flow cytometry. (c, d) Bacterial burden was determined out of spleen (c) and liver (d) homogenates. CFU numbers were normalised to organ mass. Means ± SEM are shown in the plots. (a) Survival data were compared using Cox regression and the Kaplan–Meier method using Wilcoxon’s test (Chi square 15.00, df 3, p value 0.0018.). (b, c, d) ∗p < 0.05, ∗∗p < 0.01, ∗∗∗p < 0.001 (two-way ANOVA with Tukey’s multiple comparison test. Results of the post-hoc test are presented in the plots). (b) (ns. wt low iron diet n = 21, *Tim3*^*−/−*^ low iron diet n = 19, wt high iron diet n = 27, *Tim3*^*−/−*^ high iron diet n = 30). (c) wt low iron diet n = 24, *Tim3*^*−/−*^ low iron diet n = 23, wt high iron diet n = 37, *Tim3*^*−/−*^ high iron diet n = 36. (d) wt low iron diet n = 19, wt high iron diet n1 = 19, *Tim3*^*−/−*^ low iron diet n = 29; *Tim3*^*−/−*^ high iron diet n = 34.

First, we investigated whether the deletion of TIM-3 influences the survival of mice upon *Salmonella* infection depending on the host iron status. Therefore, mice were fed with low (<9 mg elementary Fe/kg)^18^ or high iron (5 g elementary Fe/kg) diets^17^ for two weeks prior and during the systemic exposure to a lethal dose of *S. typhimurium.* Mice receiving a low iron diet had a significantly better survival than iron-supplemented mice. However, there was no difference between wt and *Tim3*^*−/−*^ littermates. Dietary iron loading resulted in a significant reduction of survival of wt mice, which became obvious after 8 days of infection whereas all iron supplemented *Tim3*^*−/−*^ mice succumbed to death within 13 days (Fig. 1a). Interestingly, the increased vulnerability to *Salmonella* infection in iron supplemented *Tim3*^*−/−*^ mice was not reflected by an altered celluarity in spleens (Fig. 1b), but was reflected by a higher bacterial burden in the spleens and livers of iron supplementend *Tim3*^*−/−*^ mice compared to all other groups. Iron supplementation in NRAMP1 expressing wt mice resulted in a significant increase of bacterial counts in spleens and a trend towards higher bacterial counts in livers (Fig. 1c and d). Supporting increased inflammation in iron supplemented *Tim3*^*−/−*^ mice, we noted a rise in weight loss and spleen index in these mice relative to wt mice (Supplementary Fig. S3a and b).

Additionally, it is well known that thrombocytopaenia is crucial during severe inflammation,^25^ therefore we measured the amounts of platelets in blood samples. No differences were seen in low iron diet fed mice but platelets were significantly reduced in iron supplemented wt mice and this reduction was exacerbated in high iron diet fed *Tim3*^*−/−*^ mice (Supplementary Fig. S3c). Next, we measured markers for metabolic disorders and organ damage. ALT/GPT activity levels were higher in high iron diet fed mice compared to low diet fed mice in both genotypes. The levels increased in high iron diet fed *Tim3*^*−/−*^ mice compared to high iron diet fed wt mice and similar effects were seen in plasma glucose values (Supplementary Fig. S3d and e). Interstingly, we found no differences in plasma LDH activity (Supplementary Fig. S3f).

Since iron is described as crucial factor for mitochondrial function we performed Seahorse analysis after 11 days of infection. CD4^+^ T cells were isolated from spleens of iron low and iron high diet fed wt and *Tim3*^*−/−*^ mice and respiration of the cells was measured. We found iron-dependent differences as the oxygen consumption rate (OCR) of iron overloaded wt and *Tim3*^*−/−*^ mice was decreased compared to low iron diet fed wt and *Tim3*^*−/−*^ mice. The OCR of high iron diet fed *Tim3*^*−/−*^ mice was lower when compared to wt. However, none of the differences were significant (Supplementary Fig. S4).

### TIM-3 controls infection-controlling molecules IFNγ and IL-10 in an iron rich environment

**Fig. 2:**
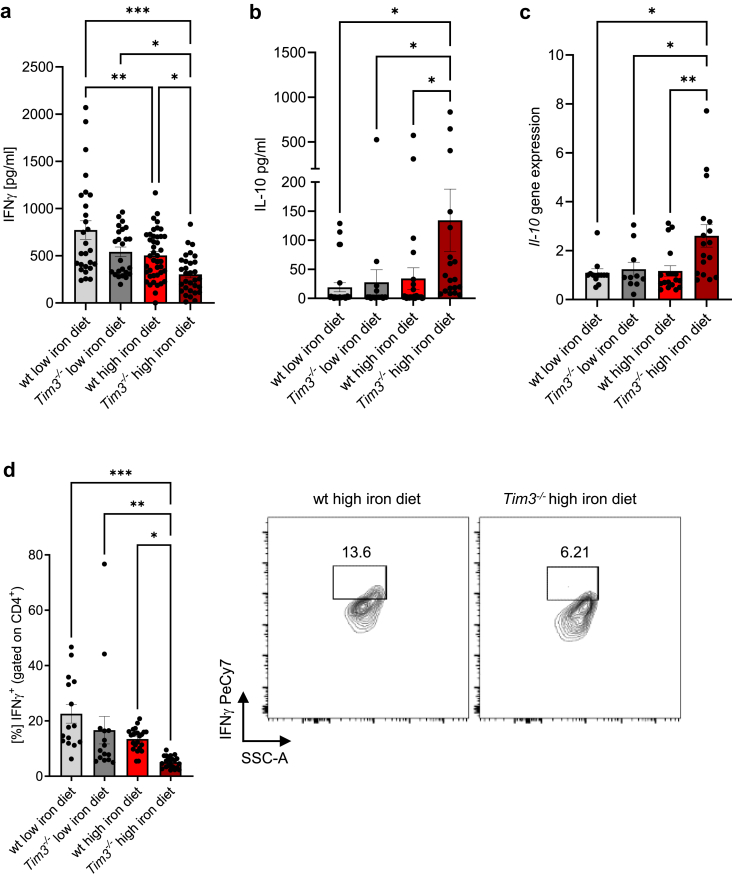
**TIM-3 is essential for the production of crucial infection-controlling molecules IFNγ and IL-10 in an iron rich environment.** Mice were fed different iron diets for two weeks prior and during the course of an intraperitoneal infection with 1000 living *S*. *typhimurium*. Serum IFNγ (a) and IL-10 (b) were measured. *Il-10* transcript levels were determined by quantitative real-time PCR and normalised to *Hprt* mRNA levels using the ΔΔCT method (c). Percentages of IFNγ-positive cells within CD4^+^ lymphocytes was quantified by flow cytometry (d). Means ± SEM are shown in the plots. ∗p < 0.05, ∗∗p < 0.01, ∗∗∗p < 0.001 (two-way ANOVA with Tukey’s multiple comparison test. Results of the post-hoc test are presented in the plots). (a) wt low iron diet n = 27, wt high iron diet n = 42, *Tim3*^*−/−*^ low iron diet n = 24,*Tim3*^*−/−*^ high iron diet n = 31. (b) wt low iron diet n = 26, wt high iron diet n = 35, *Tim3*^*−/−*^ low iron diet n = 24, *Tim3*^*−/−*^ high iron diet n = 19. (c) wt low iron diet n = 12, wt high iron diet n = 18,*Tim3*^*−/−*^ low iron diet n = 10, *Tim3*^*−/−*^ high iron diet n = 17. (d) wt low iron diet n = 15, wt high iron diet n = 23, *Tim3*^*−/−*^ low iron diet n = 15, *Tim3*^*−/−*^ high iron diet n = 22.

Since IFNγ serves as primary central cytokine in the host defence against *Salmonella*, we initially assessed IFNγ levels in plasma samples. Although, no differences could be detected in the low iron diet groups, there was a significant decrease in IFNγ levels (Fig. 2a), accompanied by a significant increase in plasma IL-10 concentrations (Fig. 2b) in iron loaded *Tim3*^*−/−*^ mice. This trend was consistent with the observed upregulation of *Il-10* gene expression in splenocytes of high iron diet fed *Tim3*^*−/−*^ mice when compared to low iron diet fed *Tim3*^*−/−*^ mice or to littermate controls (Fig. 2c).

Interestingly, the circulating levels of other central pro-inflammatory cytokines IL-1β, IL-6, IL-12 and transforming growth factor beta (TGFβ) were similar when measured in plasma samples or when analysed by gene expression analysis (Supplementary Fig. 1a–c). As the IFNγ expressing Th1 subset plays a central role in providing effective immune protection against *S. typhimurium*,^26^ we next investigated whether the decrease in IFNγ is referred to an effect of TIM-3 deletion on a particular T cell subgroup. Importantly, dietary iron loading of *Tim3*^*−/−*^ mice dramatically reduced frequencies of IFNγ producing Th1 cells within splenic helper CD4^+^ T cells on day 11-post bacterial challenge when compared to equally treated wildtype mice (Fig. 2d). Only slight differences were seen between low iron groups.

While iron loading per se reduced IFNγ producing T cells in both genotypes, the reduction was most pronounced in iron loaded *Tim3*^*−/−*^ mice (Fig. 2d). Of note, we did not observe any substantial effects of dietary iron overload on the relative and absolute numbers of Th2, Th17 and Treg lymphocytes in the spleen (Supplementary Fig. 2a). Furthermore, no significant differences were detected in relative frequencies of any of the investigated myeloid populations (neutrophils, macrophages, Ly6C^high^ classical monocytes and Ly6C^low^ resident monocytes) in association with the iron content of the diets or to the presence/absence of TIM-3 (Supplementary Fig. 2b). These findings indicate similar levels of systemic inflammation across all experimental groups.

Since TIM-3 is implicated in the development of exhausted CD8^+^ T cells in various cancer models^27^ but to a lesser extent in different models of inflammation, we analysed the expression of TIM-3, IFNγ, and IL-10 on CD8^+^ T cells in all experimental groups. In the *Salmonella* infection model, the quantity of dietary iron did not affect the expression of TIM-3, IFNγ, or IL-10 on CD8^+^ T cells (Supplementary Fig. 2c). This suggests that the effect of iron on *Tim3*^*−/−*^ mice was primarily restricted to Th1 cells.

### TIM-3 deficiency prompts IL-10 production by IL-12R-dependent CD4^+^CTL under iron-loading conditions

**Fig. 3:**
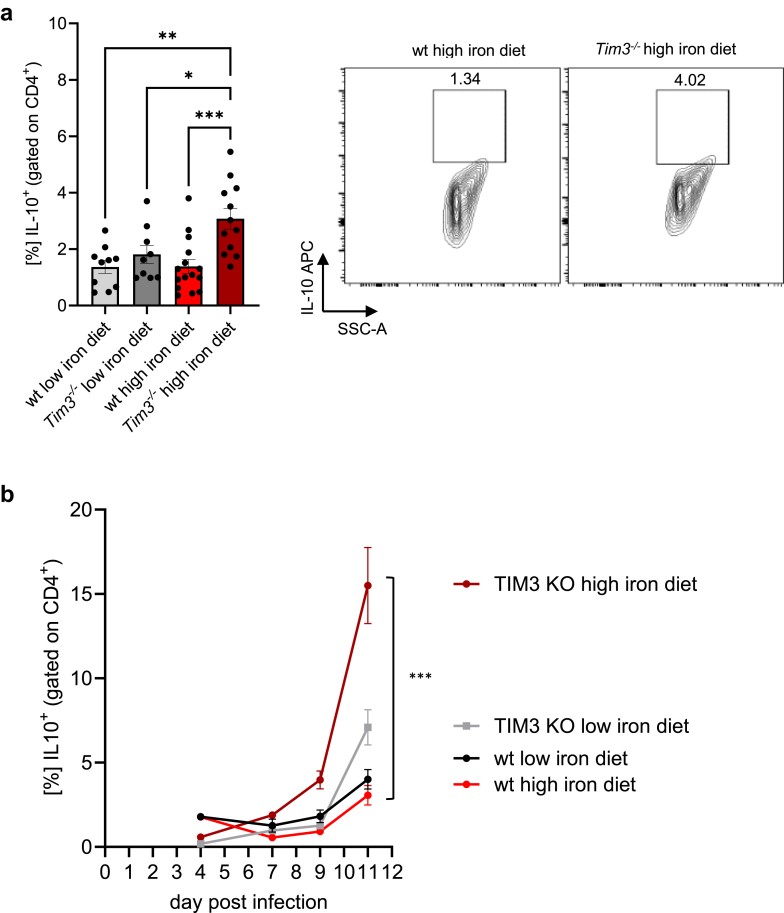
**In a setting of elevated iron levels and infection, the absence of TIM-3 signalling triggers the generation of IL-10.** Mice were fed different iron diets for two weeks prior and during the course of an intraperitoneal infection with 1000 living *S*. *typhimurium*. The percentages of IL-10-positive cells within CD4^+^ lymphocytes were quantified by flow cytometry at day 11 post-infection (a) and in time-course analysis from day 4 on until day 11 (b). Means ± SEM are shown in the plots. ∗p < 0.05, ∗∗p < 0.01, ∗∗∗p < 0.001 (two-way ANOVA with Tukey’s multiple comparison test. Results of the post-hoc test are presented in the plots). (a) wt low iron diet n = 10, wt high iron diet n = 15, *Tim3*^*−/−*^ low iron diet n = 9, *Tim3*^*−/−*^ high iron diet n = 12. (b) Day 11: wt low iron diet n = 2, wt high iron diet n = 5, *Tim3*^*−/−*^ low iron diet n = 2, *Tim3*^*−/−*^ high iron diet n = 3.

Interestingly, alongside the decreased IFNγ expression, we noted a significant rise in IL-10 levels in CD4^+^ T cells of iron loaded *Tim3*^*−/−*^ mice compared to the other groups (Fig. 3a). This consistent increase, peaking in iron overloaded *Tim3*^*−/−*^ mice, was further validated by a time course analyses starting from day 4 post-infection (Fig. 3b).

**Fig. 4:**
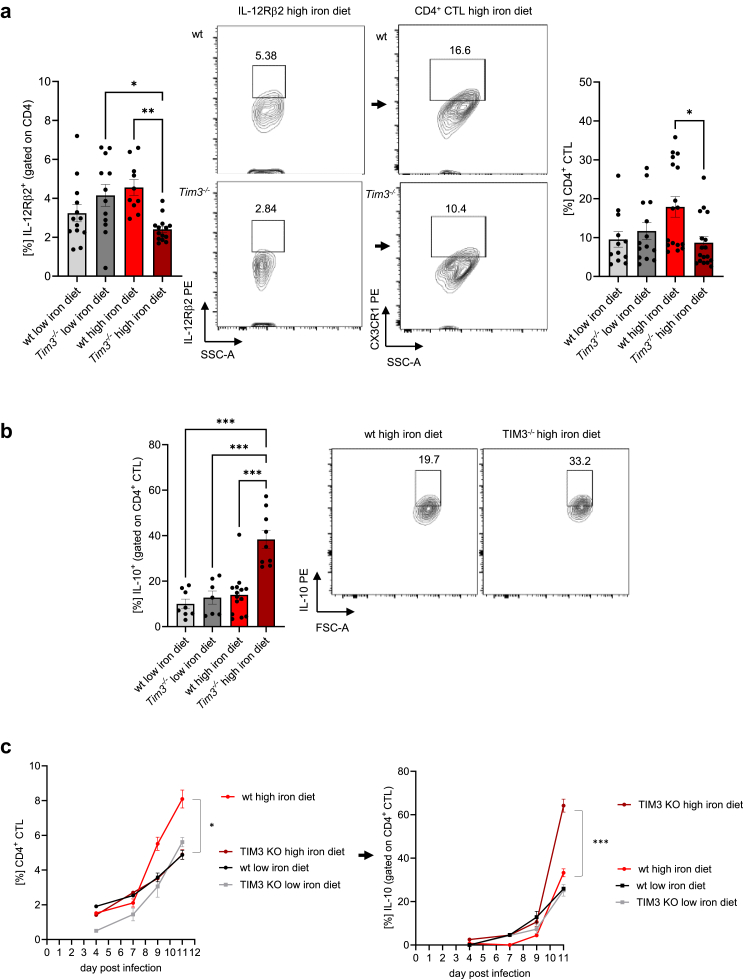
**Iron supplemented TIM-3^−/−^ cytotoxic CX3CR1^+^ IFNγ^high^ CD4^+^ T cells produce high amounts of IL-10, therefore negatively regulating the IL12Rβ2 subunit of the IL-12 receptor.** Mice were fed different iron diets for two weeks prior and during the course of an intraperitoneal infection with 1000 living *S*. *typhimurium*. (a) Percentages of IL12Rβ2 (left panel) or CX3CR1 (right panel) expressing cells within CD4^+^ helper T cells were determined by flow cytometry. (b) Percentage of IL-10 expressing CD4^+^ CTL was quantified by flow cytometry. (c) Time-course analysis for CD4^+^ CTL and IL-10 expression of CD4^+^ CTL was performed from day 4 until day 11. Means ± SEM are shown in the plots. ∗p < 0.05, ∗∗p < 0.01, ∗∗∗p < 0.001 (two-way ANOVA with Tukey’s multiple comparison test. Results of the post-hoc test are presented in the plots). (a) Left panel: wt low iron diet n = 13, wt high iron diet n = 10, *Tim3*^*−/−*^ low iron diet n = 12, *Tim3*^*−/−*^ high iron diet n = 14; right panel: wt low iron diet n = 12, wt high iron diet n = 17, *Tim3*^*−/−*^ low iron diet n = 14, *Tim3*^*−/−*^ high iron diet n = 17. (b) wt low iron diet n = 8, wt high iron diet n = 14, *Tim3*^*−/−*^ low iron diet n1 = 7, *Tim3*^*−/−*^ high iron diet n = 9. (c) Two-way ANOVA day 11: left panel: wt low iron diet n = 4, wt high iron diet n = 5, *Tim3*^*−/−*^ low iron diet n = 2, *Tim3*^*−/−*^ high iron diet n = 5. Right panel: wt low iron diet n = 3, wt high iron diet n = 3, *Tim3*^*−/−*^ low iron diet n = 3, *Tim3*^*−/−*^ high iron diet n = 3.

Recently, a study indicated that infection with *Salmonella* induces the generation of an IL-12-independent early Th1 effector cell subset, followed by a later outgrowth of IL-12-dependent Th1 cells as part of cytotoxic CD4^+^ cells (CD4^+^ CTL).^28^ To further characterise the mechanisms underlying the highly specific effect of TIM-3 on Th1 lymphocytes but not on the other CD4^+^ T cell subsets under iron loading conditions, we studied IL-12 receptor (IL12R) signalling dependend outgrowth of functional Th1 cells. When analysing the expression of the IL12R on CD4^+^ T cells by flow cytometry, we found that in iron supplemented *Tim3*^*−/−*^ mice compared to iron supplemented wildtype and low iron diet fed *Tim3*^*−/−*^ mice CD4^+^ T cells significantly expressed lower amounts of the IL-12Rβ2 chain which, together with the IL-12Rβ1 chain represent the two functional subunits of the IL-12R (Fig. 4a). The IL-12Rβ2 chain is considered as the signalling element of the receptor, and its presence in both humans and mice is largely restricted to Th1 cells.^29^^,^^30^ Furthermore, *IL-12Rβ2*^*−/−*^ mice exhibited a significant impairment of Th1 responses.^31^

Subsequently, we hypothesised that this could also impact the amount of CD4^+^ CTL. Following the gating strategy shown in Supplementary Fig. S5a and b we detected significant decreased numbers of the CD4^+^ CTL celltype when iron supplemented wildtype mice were compared to iron loaded *Tim3*^*−/−*^ mice (Fig. 4a). Importantly, these effector cytotoxic T cells produced significantly higher amounts of IL-10, which is an inihibitor of multiple antimicrobial immune effector pathways (Fig. 4b).^32^^,^^33^ This was also supported by a time course analysis (Fig. 4c).

### IL-10 neutralisation restores IFNγ expression and improves *Salmonella* control in iron-fed *Tim3*^*−/−*^ mice

Based on our previous observations, we were interested whether blocking of IL-10 could rescue the production of IFNγ in CD4^+^ T cells and therefore be beneficial for the control of the *Salmonella* infection in conditions of iron overload in *Tim3*^*−/−*^ mice.

**Fig. 5:**
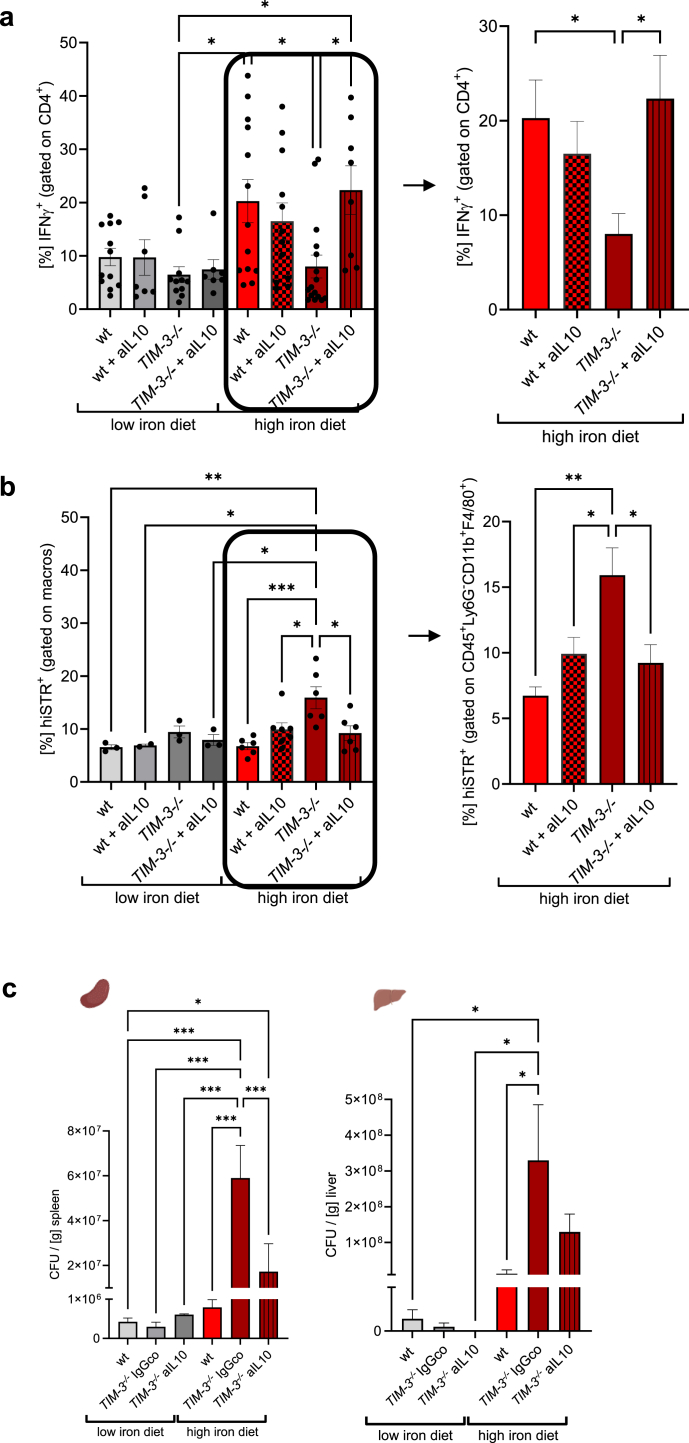
**Neutralisation of IL-10 restores IFNγ expression and pathogen control in iron supplemented *S. typhimurium*-infected *Tim3*^*−/−*^*mice*.** Mice were fed different iron diets for two weeks prior and during the course of an intraperitoneal infection with 1000 living *S*. *typhimurium*. At day 11 splenocytes were isolated and re-stimulated with RFP-expressing heat inactivated *Salmonella* and, where indicated, anti-IL-10 was added. After 48 h the percentages of IFNγ^+^ cells within CD4^+^ lymphocytes (a) and the percentage of highSTR^+^ within macrophages (CD45^+^Ly6G^−^CD11b^+^F4/80^+^) (b) were analysed by flow cytometry. (c) At day 8 post infection, mice were intraperitoneally injected once with 100 μg inVivo MAb anti-mouse IL-10 antibody or InVivoMAb rat IgG1 isotype control. The animals were analysed on day 11 post infection. Bacterial burden was determined out of spleen and liver homogenates. CFU numbers were normalised to organ mass. Means ± SEM are shown in the plots. ∗p < 0.05, ∗∗p < 0.01, ∗∗∗p < 0.001 (two-way ANOVA with Tukey’s multiple comparison test. Results of the post-hoc test are presented in the plots). (a) Low iron diet wt n = 12, wt + aIL10 n = 7, TIM3^−/−^ n = 11, TIM3^−/−^ + aIL10 n = 7; high iron diet wt n = 13, wt + aIL10 n = 12, TIM3^−/−^ n = 16, TIM3^−/−^ + aIL10 n = 8. (b) Low iron diet wt n = 3, wt + aIL10 n = 3, TIM3^−/−^ n = 3, TIM3^−/−^ + aIL10 n = 3; high iron diet wt n = 6, wt + aIL10 n = 7, TIM3^−/−^ n = 6, TIM3^−/−^ + aIL10 n = 6. (c) Left panel: low iron diet wt n = 15, TIM3^−/−^ IgGco n = 7, TIM3^−/−^ aIL10 n = 3; high iron diet wt n = 12, TIM3^−/−^ IgGco n = 3, TIM3^−/−^ aIL10 n = 5. Right panel: low iron diet wt n = 3, TIM3^−/−^ IgGco n = 3, TIM3^−/−^ aIL10 n = 3; high iron diet wt n = 5, TIM3^−/−^ IgGco n = 5, TIM3^−/−^ aIL10 n = 5.

First, we established an *ex vivo* model where splenocytes from the different treatment groups at day 11 post-infection were isolated and cultivated on cell culture inserts. Cells were restimulated with heat inactivated Red Fluorescent Protein (RFP)-expressing *Salmonella* and an IL-10 blocking antibody was added where indicated. After 72 h, splenocytes were harvested and analysed for the expression of IFNγ and for RFP expression in Ly6G^−^CD11b^+^F4/80^+^ macrophages. The addition of the anti-IL-10 blocking antibody restored IFNγ production by CD4^+^ T cells in cultured splenocytes from *Tim3*^*−/−*^ mice with iron supplementation to the level observed in wildtype mice with iron supplementation (Fig. 5a). Accordingly, re-exposure of cells to RFP-expressing heat inactivated *Salmonella* showed a significant lower amount of engulfed bacteria in macrophages of anti-IL-10 treated iron supplemented *TIM-3*^*−/−*^ splenocytes (Fig. 5b). These findings indicate that in the absence of the negative immune checkpoint regulator TIM-3, blocking of IL-10 leads to increased levels of IFNγ and better control of *Salmonella* infection similar to that observed in wildtype mice fed a high iron diet.

Next we analysed whether administering anti-IL-10 could enhance the immune response to *Salmonella* and improve infection control in iron-overloaded *Tim3*^*−/−*^ mice. At day 8 post-infection anti-IL-10 was injected in *Tim3*^*−/−*^ mice once. At day 11 post bacterial challenge organs were harvested and *Salmonella* colony forming units (CFU) were counted in spleens and livers. Importantly, the administration of the anti-IL-10 antibody caused a strong reduction in *Salmonella* CFU in iron loaded *Tim3*^*−/−*^ mice compared to the IgG isotype treated control (Fig. 5c). The efficacy of the antibody was further confirmed by the observation of a significant reduction of plasma IL-10 concentrations after the application of the anti-IL-10 antibody (Supplementary Fig. S3g).

## Discussion

Here we report a novel role for TIM-3 in the control of a *S. enterica* serovar *typhimurium* infection by T cells. Specifically, we found that under excess iron conditions the lack of TIM-3 signalling led to reduced survival of mice, which was parallelled by increased bacterial numbers in spleens and livers. In regard to immune regulation we found that iron loaded *Tim3*^*−/−*^ mice have an impaired IL-12R dependent production of the pro-inflammatory cytokine IFNγ, which could be caused by the strong upregulation of anti-inflammatory IL-10. Earlier studies indicate that in a model of prolonged bacterial infection and in a model of mouse mammary carcinomas iron has strong and highly specific effects on IFNγ producing T cells.^17^^,^^34^ At the same time, however, this appears contradictory to one of our recent studies, which demonstrated that antibody mediated blocking of TIM-3 in iron fed wildtype mice in an inflammation model led to an increase of Th1 function followed by a decrease of bacterial numbers in spleens and livers.^17^ TIM-3 was originally identified on terminally differentiated Th1 cells and subsequent research has highlighted its significant involvement in T cell dysfunction and exhaustion. This process is in part characterised by a decreased ability to release pro-inflammatory cytokines such as IFNγ.^13^^,^^14^^,^^35^ However, the function of TIM-3 and the effect of its silencing is multifaceted and also depends on the models used. On the one hand, blocking TIM-3 exacerbates disease outcome in various preclinical disease models of multiple sclerosis, inflammatory bowel disease, or diabetes, among others by hyperactivating Th1 cell immune function.^36^^,^^37^ On the other hand, in a mouse model of head and neck cancer the blockade of TIM-3 reduced tumour growth. Additionally, in *M. tuberculosis* infected mice the *in vivo* administration of a TIM-3-immunoglobulin fusion protein led to a reduction of bacterial burden.^38^ Notably, interaction of a protein with a specific antibody may result in unintended side effects due to non-specific blocking or residual protein activity caused by incomplete functional neutralisation of the target structure. When knockout mouse models are accessible, these adverse effects could be mitigated or eliminated.

In the bacterial infection model described herein the genetic deletion of TIM-3 and thus its absence on T cells have a pivotal impact for the control of the *Salmonella* infection under high iron conditions. In a previous study we investigated how dietary iron supplementation affects T cell function and outcome in the same infection model.^17^ In this study only the high iron diet fostered bacterial burden and reduced differentiation of CD4^+^ Th1 cells and expression of IFNγ. This effect could be traced back to iron-mediated induction of TIM-3 on the surface of this T cell subset. Additionally we demonstrated that *in vitro* experiments only iron supplementation specifically upregulated mRNA and protein expression of TIM-3 in naïve Th cells in a dose-dependent manner and hindered priming of those T cells towards Th1 differentiation. The detrimental effects of high systemic iron on the integrity of bacterial immune defence in this model of *Salmonella* infection could be traced back to a strong iron-mediated upregulation of TIM-3 expression in helper T cells and an impaired differentiation into protective IFNγ producing Th1 lymphocytes.

Additionally, recent findings have shown that the iron-binding ferritin H chain (FTH) plays a crucial role in promoting disease tolerance during sepsis by preventing fatal hypoglycemia triggered by acute infection^39^ which in part can be referred to modulation of regulatory T-cell function.^40^ Moreover, macrophage ferritin H protects from an inflammasome driven pathologic inflammation in infections and from adverse outcomes. Disrupted glucose metabolism plays a key role in driving the metabolic and energy failure involved in the progression of sepsis.^41^ This is reflected by most increased glucose levels and more advanced liver damage, as reflected by higher ALT/GPT levels, in high iron diet fed *Tim3*^*−/−*^ mice compared to all other groups.

Our findings raise the possibility that a significant increase of IL-10 is the crucial factor which downregulates IFNγ and therefore negatively influences the defence against *Salmonella* due to increased iron levels. Of note, IL-10 is described as one of the master-anti-inflammatory cytokines. However, Zhu et al. demonstrated that another immunosuppressive cytokine, IL-27, induces TIM-3 and IL-10 expression in activated T cells via the transcription factor Nuclear factor, interleukin 3 regulated (NFIL-3).^42^ This simultaneous activation of TIM-3 and IL-10 might suggest reduced IL-10 levels in TIM-3 knockout mice. However, compared to the gut inflammation model described in the publication by Zhu et al., the *Salmonella* sepsis model used by us is a more generalised inflammation model affecting multiple immune cells and signalling pathways. Moreover, adding excess amounts of iron during an infection with *Salmonella* influences a multitude of metabolic pathways, pointing in the direction that a reverse conclusion concerning IL-10 expression in *Tim3*^*−/−*^ mice should be drawn cautiously. Of note, IL-27 is a cytokine with reported immunostimulatory and immunosuppressive effects.^43^ However, to analyse whether and how the pathway via IL-27 and NFIL-3 is of importance in the defence against *Salmonella* in an environment of iron excess and in the absence of TIM-3 signalling should be part of future investigations.

In agreement with the strong decrease of IFNγ we found that the increase of IL-10 on CD4^+^ T cells in *Salmonella* infection is time dependent with the highest levels in iron fed *Tim3*^*−/−*^ mice. This is likely functionally linked, because IL-10 is an efficient inhibitor of pro-inflammatory immune effector functions including IFNγ formation.^44^ In addition, iron supplementation can augment IL-10 formation in mouse *Salmonella* sepsis models.^45^ These findings raised the question whether a specific subgroup of CD4^+^ T cells could be responsible for our observations. In mice and humans a Th subset with MHCII - dependent cytotoxic activity has recently become apparent.^46^ These CD4^+^ cytotoxic T lymphocytes are highly differentiated, antigen-experienced cells, with T-bet and Eomes serving as key upstream transcription factors driving their cytolytic function.^47^ While some CD4^+^ CTL may originate from Th1 cells, other subsets exhibit transcriptional profiles that are distinct from Th1 cells. Their ability to produce IFNγ, perforin, and granzyme sets them apart from conventional CD4^+^ T cells, reflecting the cytotoxic role of CD8^+^ T cells and allowing them to directly eliminate target cells. CD4^+^ CTL were also found in the peripheral blood of patients suffering from various viral infections, autoimmune diseases, or cancer where CD4^+^ T cells are exposed to antigens over a longer period of time.^48^^,^^49^ However, there is limited information on the role of CD4^+^ CTL in the defence against bacterial infections. Recent evidence indicated that during infection with *S. enterica* Th1 cells formed a cytotoxic subset, which controlled the bacterial multiplication in an interleukin-12 (IL-12) dependent manner.^28^ Another study described CD4^+^ CTL as important subset during murine *Brucella abortus* infection. CD4^+^ CTLs had cytolytic capabilities against *Brucella*-infected but not against *Mycobacterium*-infected APCs. Furthermore, in peripheral blood mononuclear cells (PBMC) from tuberculosis patients, blocking of IL-10 resulted in higher lytic activity of CD4^+^ and CD8^+^ cells. Importantly, the supplementation of IL-10 during CTL induction restrained the cells’ capability to lyse macrophages, which were pulsed with γ-irradiated *M. tuberculosis*.^50^

In infected, iron overloaded *Tim3*^*−/−*^ mice we identified CD4^+^ CTL as producers of high IL-10 levels. This aligns with recently published data, which describe that during an infection with *S. enterica* Th1 cells developed into a cytotoxic subgroup that could effectively manage the infection through an IL-12 dependent pathway.^28^ Our data support the crucial role of IL-12 signalling for the function of CD4^+^ CTL. Krueger et al. demonstrated that upon *Salmonella* infection Th1 cells undergo IL-12-dependent differentiation leading to the generation of CD4^+^ CTL. Our data did not show any iron and TIM-3 signalling dependent changes in IL-12 plasma concentrations, but the amount of the IL-12Rβ2 chain, which is the central signalling component of the IL-12R driving differentiation of CD4^+^ towards a Th1 phenotype was strongly reduced in iron fed *Tim3*^*−/−*^ mice. Apart from TIM-3, iron may contribute to this phenotype as it was shown to impact on STAT-1 mediated signalling pathways.^51^ This could be the reason for the decreased amounts of CD4^+^ CTL. CD4^+^ CTL express several markers characteristic for Th1 cells such as IFNγ, thus a dysfunctional IL-12 pathway could be a part of the mechanism leading to the exaggerated IL-10 production by these cells.^52^^,^^53^ Moreover, iron has been shown to increase IL-10 formation mainly by macrophages in several *in vitro* and *in vivo* models and can thus ameliorate overwhelming IL-10 formation in the absence of TIM-3.^45^^,^^54^ Although blocking of IL-10 *in vivo* and *ex vivo* led to a better *Salmonella* infection control in our model, the complex interplay of iron excess and the absence of TIM-3 signalling and their influence on the function of CD4^+^ CTL warrants further investigation.

Iron loading is a frequent clinical condition in subjects suffering from genetic defects including haemochromatosis or hemoglobinopathias but also in patients who received multiple blood transfusion f. e. in the course of surgical procedures or hemato/oncological therapies including stem cell transplantation. Many of these patients are at a higher risk for a high infection related morbidity and mortality which can be further increased by immune suppression due to the underlying disease or immune-suppressive medication.^55^ Importantly, many patients suffering from inflammatory diseases or cancer suffer from functional iron deficiency, which largely results from inflammation and hepcidin driven iron retention in macrophages making iron less available for erythropoiesis thereby contributing to the development of anaemia of inflammation.^56^ It is thus important to correctly diagnose alterations of iron homoeostasis and the concomitant cause of anaemia in patients with inflammatory diseases. While iron supplementation is likewise essential in the setting of absolute iron deficiency with empty iron stores its effeciacy in the setting of functional iron deficiency and anaemia of inflammation is questionsable, because supplemented iron is retained in the reticuloendothelial system (RES) or not properly absorbed in the duodenum, specifically under conditions with more sustained inflammation.^57^^,^^58^ Based on the notion, that iron is also an essential nutrient for many microbes but also on the fact that iron impacts immune function as shown herein, unbiased iron supplementation to patients with inflammatory disease and functional iron deficiency can affect anti-microbial immune effector pathways and promote pathogen expansion.^59^^,^^60^ Gaining new knowledge on the effects of immune regulators and iron on the immune control of infection can pave the way to improved treatment of such infection in an era of exaggerating antimicrobial resistance.

Our data uncover an essential role of the checkpoint inhibitor TIM-3 for the control of bacterial infections, specifically under iron overloading conditions. We demonstrate that during iron loading conditions TIM-3 controls IL-10 formation and improves Th1 mediated IFNγ formation leading to better control of *Salmonella* infection. This places TIM-3 at the forefront of an innovative mechanism through which iron influences the functionality of CD4^+^ cells, offering fresh insights into the pathophysiology of bacterial septicaemia by highlighting the interconnected relationship between iron and T cell functions. Hence, TIM-3 could emerge as a promising therapeutic target for addressing chronic infections caused by intracellular pathogens, particularly in conditions characterised by an excess of iron. Nonetheless, a key limitation of the study is that TIM-3 is not exclusively expressed on T cells. It is also found on several innate immune cells such as macrophages, dendritic cells, and monocytes. These innate cells can significantly shape the immune response by presenting antigens, producing cytokines, and regulating the inflammatory environment, thereby indirectly influencing T cell activation, differentiation, and function. To clarify whether the immunological effects observed under conditions of iron supplementation and bacterial infection are specifically mediated by TIM-3 signalling in T cells or are partly driven by TIM-3 activity in innate immune populations further studies including the analysis of T cell conditional TIM-3 knockout mice will be needed.

## Contributors

CPO participated in the study design, data collection, and analysis, and drafted the manuscript. NB participated in the study design and revised the manuscript. PT, ED, CV, and SE participated in the data collection and revised the manuscript. GW participated in the study design, data collection, analysis, data interpretation, and funding and manuscript preparation. Data were assessed and verified by CPO, NB, PT, ED, CV, and GW. CPO and GW were responsible for the decision to submit the manuscript. All authors read and approved the final manuscript.

## Data sharing statement

The data that support the findings of this study are available in the manuscript or in the supplementary materials upon publication or upon request from corresponding authors.

## Declaration of generative AI and AI-assisted technologies in the writing process

During the preparation of this work the author(s) used ChatGPT in order to improve language and readability. After using this tool/service, the author(s) reviewed and edited the content as needed and take(s) full responsibility for the content of the publication.

## Declaration of interests

The authors declare that the research was conducted in the absence of any commercial or financial relationships that could be constructed as a potential conflict of interest.
