# Supplementary material for: TIM-3 ameliorates host responses to Salmonella infection by controlling iron driven CD4+ T cell differentiation and interleukin-10 formation
Source: eBioMedicine. 2025 Sep 11;120:105910. doi: 10.1016/j.ebiom.2025.105910 (PMC12571576; doi:10.1016/j.ebiom.2025.105910)
Supplement: Supplementary Figs. S1–S5 [file mmc1.docx]

**SUPPLEMENTARY MATERIAL**

**TIM-3 ameliorates host responses to *Salmonella* infection by controlling iron driven CD4^+^ T cell differentiation and interleukin-10 formation**

Christa Pfeifhofer-Obermair^1^*, Natascha Brigo^1^, Chiara Volani^1^, Piotr Tymoszuk^1^, Egon Demetz^1^, Sabine Engl^1^, and Günter Weiss^1,2^*

^1^Department of Internal Medicine II, Medical University of Innsbruck, Innsbruck, Austria; ^2^Christian Doppler Laboratory for Iron Metabolism and Anemia Research, Medical University of Innsbruck, Innsbruck, Austria.

***Corresponding authors:**

Christa Pfeifhofer-Obermair, PhD and Günter Weiss, MD Medical University of Innsbruck, Department of Internal Medicine II, Anichstr. 35, Innsbruck, Austria;

[christa.pfeifhofer@i-med.ac.at](mailto:christa.pfeifhofer@i-med.ac.at) or [guenter.weiss@i-med.ac.at](mailto:guenter.weiss@i-med.ac.at);

telephone number: +43 (0)512/504-23251

**
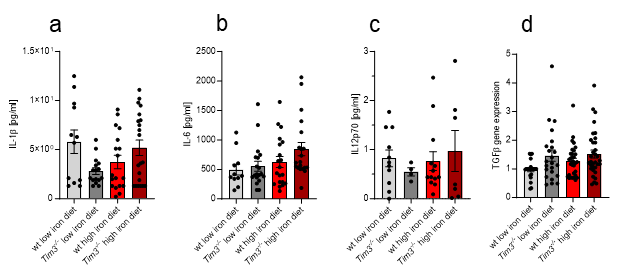
**

**Supplementary Figure 1.**

**
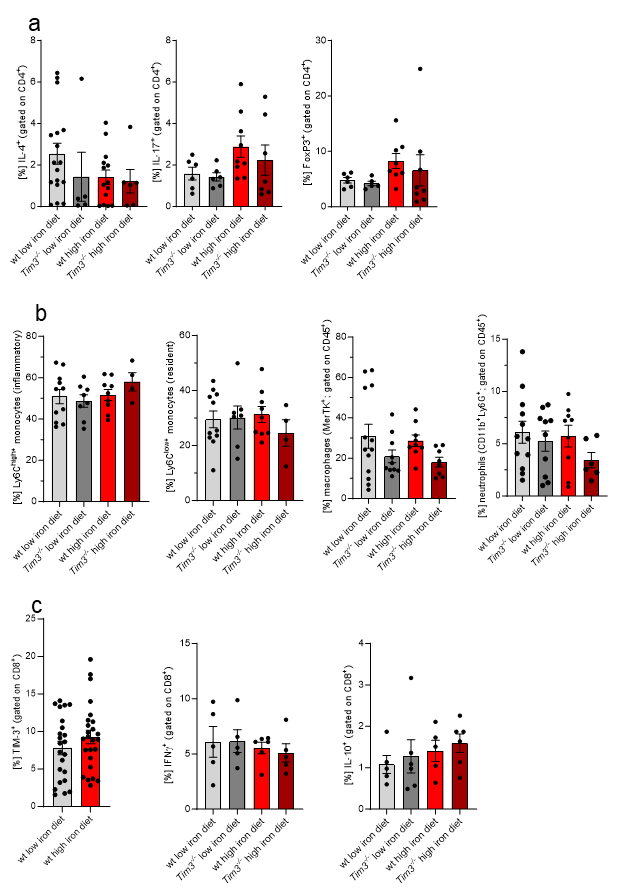
**

**Supplementary Figure 2.**


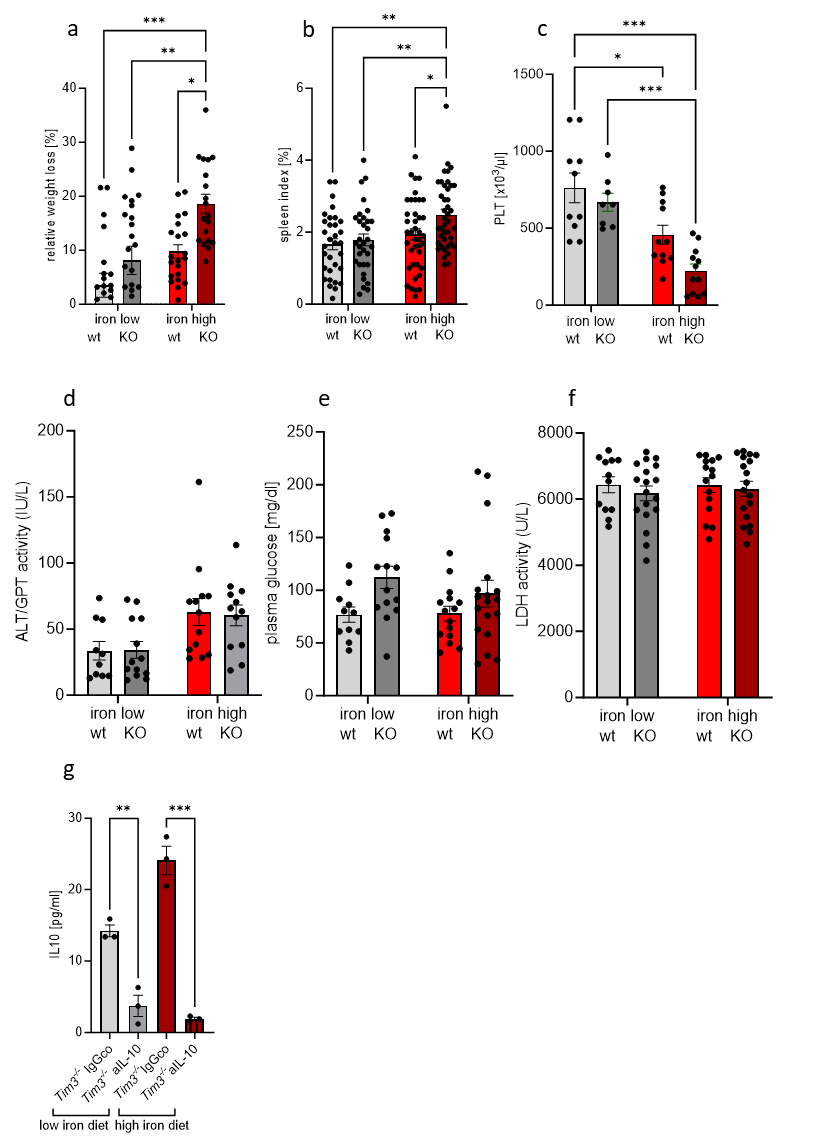


**Supplementary Figure 3.**

**Supplementary Figure 4.**


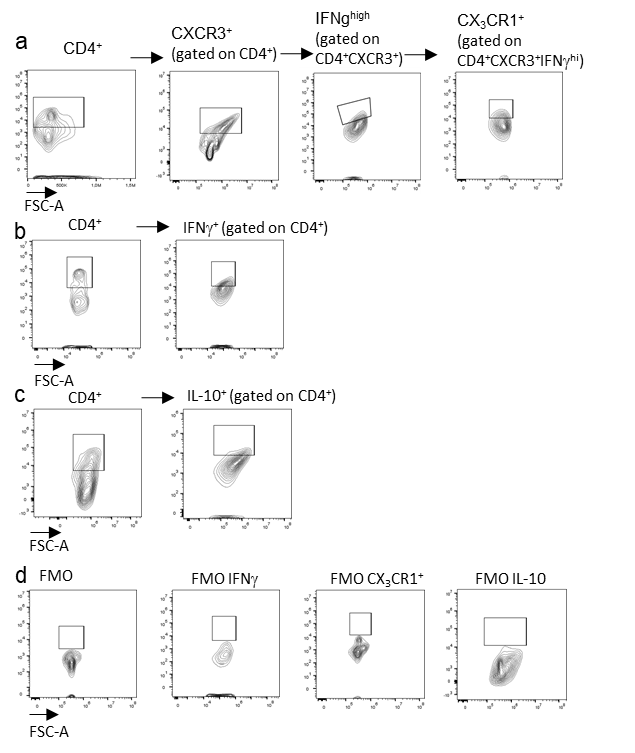


**Supplementary Figure 5.**
